# KIF11 and KIF14 Are a Novel Potential Prognostic Biomarker in Patients with Endometrioid Carcinoma

**DOI:** 10.3390/cancers17050804

**Published:** 2025-02-26

**Authors:** Paulina Antosik, Justyna Durślewicz, Marta Smolińska-Świtała, Jonasz Podemski, Edyta Podemska, Izabela Neska-Długosz, Jakub Jóźwicki, Dariusz Grzanka

**Affiliations:** Department of Clinical Pathomorphology, Faculty of Medicine, Collegium Medicum in Bydgoszcz, Nicolaus Copernicus University in Toruń, 85-094 Bydgoszcz, Poland; justyna.durslewicz@op.pl (J.D.); marta_smolinska@op.pl (M.S.-Ś.); j.podemski91@gmail.com (J.P.); edyta.trbk@gmail.com (E.P.); jakubj88@gmail.com (J.J.); d_grzanka@cm.umk.pl (D.G.)

**Keywords:** endometrial carcinoma, kinesins, KIF11, KIF14, prognostic biomarker

## Abstract

**Background** The current study aimed to evaluate the function of KIF11 and KIF14 in endometroid cancer and assess their role as a prognostic biomarker and therapeutic target for treating endometroid cancer. **Methods** Therefore, we immunohistochemically tested KIF11 and KIF14 proteins in tumoral and non-tumoral tissue sections from *n* = 92 endometroid cancer patients with respect to major prognostic and predictive characteristics, as well as treatment outcome. **Results** We found that KIF11 protein levels were higher in tumor tissues as compared to normal tissues, whereas KIF14 protein levels were lower in cancer tissue. High KIF11 protein levels and low KIF14 protein levels were independent prognostic factors for poor OS. Furthermore, the expression profile of KIF11highKIF14low was associated with the worst OS, and it was superior to each single protein alone in predicting patient prognosis. **Conclusions** In summary, individual and combined expression of KIF11 and KIF14 proteins may serve as potential prognostic biomarkers for endometroid cancer.

## Simple Summary

Endometrioid carcinoma (EC) is one of the most prevalent gynecological cancers, with rising incidence and mortality rates. Identifying reliable prognostic biomarkers is crucial for improving patient outcomes. This study investigates the role of KIF11 and KIF14 in EC prognosis. Immunohistochemical analysis of 92 tumor samples showed that high KIF11 and low KIF14 expression are independent indicators of poor overall survival. Notably, the combined KIF11highKIF14low expression pattern was associated with the worst prognosis, surpassing the predictive value of each marker alone. These findings suggest that KIF11 and KIF14 expression levels, particularly when analyzed together, could serve as valuable prognostic biomarkers, aiding in risk stratification and potentially guiding targeted therapeutic approaches for EC patients. This study investigates the prognostic significance of KIF11 and KIF14 in endometrioid carcinoma, focusing on their potential as biomarkers and therapeutic targets. Using immunohistochemical analysis of 92 tumor samples, we demonstrated that high KIF11 and low KIF14 expression correlate with poor patient outcomes, suggesting their combined evaluation offers superior prognostic value.

## 1. Introduction

Globally, endometrial carcinoma (EC) is the sixth most frequently diagnosed cancer in women and a predominant gynecologic malignancy [1]. The highest number of cases is observed in highly developed countries, especially in the United States and Europe [2,3,4,5]. Moreover, the incidence of EC continues to increase. This is likely due to increased life expectancy, high obesity rates, changes in female reproductive patterns, and numerous other risk factors [2,6,7,8]. EC is diagnosed in 80% of postmenopausal women, although it also affects very young women [7]. The average age at diagnosis is 60–63 years [8]. According to the International Agency for Research on Cancer, the estimated number of new cases in 2040 will increase from 417,367 (2020) to 543,710, i.e., by 30.3%, and the heath rate related to EC will increase from 97,370 (2020) to 145,843 i.e., by 49.8% [9]. Factors that contribute to the risk of EC include the over exposure of the endometrium to estrogens, early first menstruation, infertility, or an ovulation, polycystic ovary syndrome, or late-life menopause, obesity, and metabolic syndrome.

Since 1983, two main types of EC have been distinguished. Type I (endometrioid) is the highest percentage and reaches 70–90% of all cases. It is closely related to excessive estrogenic stimulation. Type I tumors are diagnosed mainly in young women and in perimenopausal women and are characterized by a low degree of malignancy and generally have a favorable prognosis. They are formed against the background of hyperplasia. Type II (non-endometrioid) accounts for a smaller percentage of cases; however, due to its more aggressive course, as many as 40% of cases are fatal. Detected mainly in elderly postmenopausal women, the pathogenesis of type II tumors is not estrogen-dependent, and the risk of recurrence and metastasis are very high. In 2013, through The Cancer Genome Atlas TCGA project), four additional metabolic subgroups of EC were distinguished: ultra-mutated EC with mutations in the DNA exonuclease domain-epsilon polymerase (POLE); type mutated EC with microsatellite instability; high-copy EC with frequent TP53 mutations; and a group of low copy endometria tumors. It has been shown that women in the subgroup with the POLE mutation have the best chance of survival, while women in the subgroup with the high copy number have the worst prognosis [3,4,5,10,11]. EC profiling and metabolic classification are expected to contribute to the development of targeted therapy and a more individualized approach to the patient [3,4,11]. Classical therapies are burdened with numerous side effects; therefore, the search for new markers that could become potential targets for modern pharmacotherapy has become extremely important [8,12,13].

The kinesin superfamily (KIF) is a group of proteins that play a role in many physiological processes, such as intracellular transport, mitosis, meiosis, and movement. They are also defined as motor proteins that transport organelles, mRNA, and protein complexes along microtubules in an ATP-dependent manner. The KIF superfamily can be divided into 14 families (named kinesin-1 through kinesin-14), among which the latter proteins have been shown to play different roles in the pathobiology of various human cancers [14].

Kinesin-3-KIF14 and kinesin-5-KIF11 are encoded by genes located on chromosome 1 locus 1q31.1 and chromosome 10 locus 10q24.1, respectively [14]. KIF14 exhibited a presence along the spindle midzone throughout mitosis; however, it displayed pronounced enrichment specifically at the midbody during cytokinesis. Whereas KIF11 is a plus-end directed kinesin that plays an important role through segregation and bipolar spindle assembly in metaphase [15]. Both kinesins have been proposed to have non-mitotic functions as well, e.g., in regulating axonal branching, growth cone motility, and cell motility [15]. It is, therefore, not surprising that abnormal expression of these proteins may lead to disorders of centrosome separation, cytokinesis, and bipolar spindle assembly, which may be one of the pathways for the development of many cancers [16]. KIF14 or KIF11 are overexpressed in multiple tumor types including ovarian cancer, breast cancer, and EC [17,18,19,20]. Importantly, this overexpression has clinical relevance: high KIF14 or KIF11 expression is associated with poor patient survival time. It draws our attention to the importance of KIF14 or KIF11 as a prognostic factor. Therefore, the aim of our article was to investigate the prognostic value of the two kinesins’, KIF11 and KIF14, protein levels as individual and combined biomarkers for EC patients.

## 2. Materials and Methods

### 2.1. Patients and Tissue Specimens

This study was performed on formalin-fixed paraffin-embedded tissue (FFPE) specimens from patients with EC who were diagnosed in Jan Biziel University Hospital No. 2 in Bydgoszcz. The study material consisted of EC tumor samples from 92 patients who underwent total abdominal hysterectomy with bilateral uterine appendages and pelvic lymph node dissection. The clinical staging was estimated based on the evaluation of postoperative tissue material by two independent pathologists (D.G. and J.J.) according to the International Federation of Gynecology and Obstetrics (FIGO) system and the TNM classification of malignancies (TNM). Postoperative survival data were available for all patients. The control group included material collected from 45 patients who underwent hysterectomy due to uterine fibroids. Histopathological assessment of the control material confirmed the presence of the normal endometrial glandular epithelium, and no inflammatory changes or dysplasia were found in the analyzed tissues.

This study was approved by the Bioethical Commission of Collegium Medicum in Bydgoszcz of the Nicolaus Copernicus University in Torun, Poland (decision: No KB 87/2020).

### 2.2. Tissue Samples

This study was conducted on tissue macroarrays constructed from the representative tumor areas. Tissue fragments from donor blocks, each containing samples from five patients with endometrial cancer, were meticulously transferred to pre-prepared acceptor blocks. The selected tissue was then re-embedded in paraffin to create recipient blocks, which were subsequently sectioned into 4-µm-thick slices using a rotary microtome (Accu-Cut^®^ SRM™ 200; Sakura, Torrance, CA, USA). The prepared sections were mounted on extra-adhesive slides (Superfrost Plus; Mensel-Glazer, Braunschweig, Germany) and incubated on a heating plate at 60 °C for one hour to ensure optimal adhesion and sample integrity.

### 2.3. Immunohistochemistry

Immunohistochemical (IHC) staining was performed on the BenchMark^®^ ULTRA system (Roche Diagnostics/Ventana Medical Systems, Tucson, AZ, USA). The analysis utilized a rabbit polyclonal anti-KIF11 antibody (cat. no: HPA010568, Sigma-Aldrich, St. Louis, MO, USA) at a 1:150 dilution and a rabbit polyclonal anti-KIF14 antibody (cat. no: HPA038061, Sigma-Aldrich, St. Louis, MO, USA) at a 1:500 dilution. The reaction was detected using the visualization system (Ultra View DAB Detection Kit; Ventana Medical System, AZ, USA) according to a previously approved protocol. A series of positive and negative control reactions were performed to standardize the immunohistochemical procedures. KIF11 positive control was performed on lymph node tissue, and KIF14 on urinary bladder showing the presence of the antigens analyzed in the reference sources (The Human Protein Atlas: https://www.proteinatlas.org, accessed on 25 September 2021), as well as in the data sheet of the antibody produced. A negative control was performed by replacing the primary antibody with phosphate-buffered saline.

### 2.4. Expression Analysis

Evaluation of protein expression was carried out at 20x original objective magnification for each of the studied antibodies in five places, with representative expression so-called hot spots. The protein expression was estimated using morphometric rules based on the Remmele–Stegner scale, according to ratio of the intensity of protein expression (0—negative, 1—low expression, 2—moderate expression, 3—strong expression) and percentage of the positively staining number of cancer cells in the study group and endometrial glandular cells in the control group ((0) negative; (1) ≤10% positive area; (2) 11–50% positive area; (3) 51–80% positive area; (4) >80% positive area). Pathologists who evaluated the immunohistochemical expression of the tested antigens worked independently using an Olympus BX53 light microscope (Olympus, Tokyo, Japan). The final staining result was divided into two expression groups based on a specific discrimination cutoff point determined by Evaluate Cutpoints software (R version 3.4.1) [21]. The cutpoint values for low and high expression of KIF11 and KIF14 were as follows: <1; ≥1, <3; ≥3, respectively. Moreover, we established the combination of expression of the examined factors. KIF11^high^KIF14^low^ cases were analyzed in comparison with the opposite expression pattern, KIF11^low^KIF14^high^.

### 2.5. Statistical Analysis

All statistical analyses were conducted using SPSS software version 26.0 (IBM Corporation, Armonk, NY, USA) and GraphPad Prism version 8.0 (GraphPad Software, San Diego, CA, USA). The Shapiro–Wilk test, Mann–Whitney test, Fisher’s exact test, and Chi-squared test were applied as appropriate. Survival analysis was performed using the Kaplan–Meier method with a log-rank test. To assess the correlation between KIF11 and KIF14 expression levels and clinical characteristics, univariate and multivariate logistic regression models were employed. A significance threshold of *p* < 0.05 was applied to determine statistical significance.

## 3. Results

### 3.1. Patient Characteristics of the Study Cohort

Histologically, only patients with endometrioid carcinoma were included in this study. The overall median age was 66 years (ranging from 40 to 84 years). Regarding the tumor differentiation, there were 7 (7.61%), 60 (65.22%), and 25 (27.17%) cases ranging from G1, G2, to G3, respectively. In the present study, there were 54 (58.70%) cases of pT1, 22 (23.91%) cases of pT2, 13 (14.13) cases of pT3, and 3 (3.26%) cases of pT4. Out of 92 patients, 13 (14.13%) were found to have lymph node metastases, whereas 79 (85.87%) had no evidence of lymph node involvement (N0). Eighty-four (91.30%) patients were M0 and eight (8.70%) patients were M1. FIGO I, II, III, and IV comprised 48 (52.17%), 18 (19.57%), 18 (19.57%), and 8 (8.70%), respectively. The median follow-up time was 122 months. The clinicopathologic characteristics of the patients are shown in Supplementary Table S1.

### 3.2. Comparison of Protein Expression Levels Between Cancer and Normal Tissues in Our Own Cohort and Evaluation of Its Relationship with Clinical-Pathological Features

**Figure 1:**
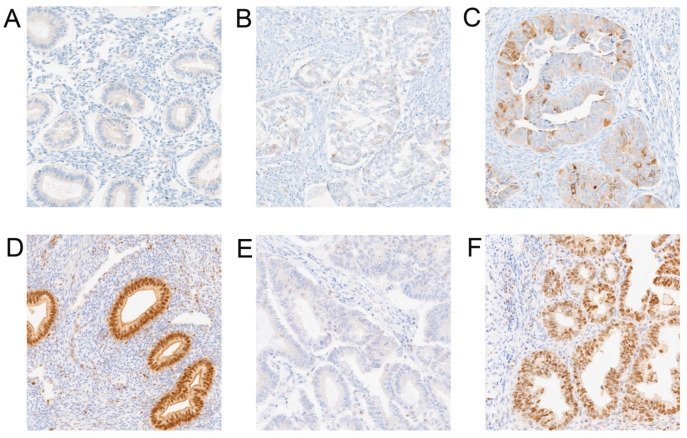
Representative images of immunohistochemistry staining for KIF11 and KIF14 within normal and endometrium cancer tissues (primary magnification ×20). (**A**) KIF11 representative staining in control; (**B**) KIF11 weak staining in endometrial cancer; (**C**) KIF11 strong staining in endometrial cancer; (**D**) KIF14 representative staining in control; (**E**) KIF14 weak staining in endometrial cancer; (**F**) KIF14 strong staining in endometrial cancer.

**Figure 2:**
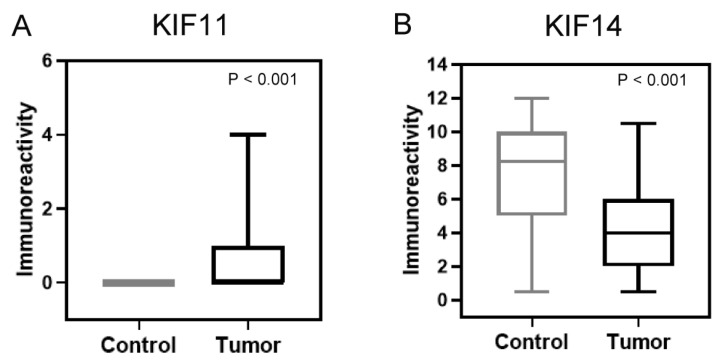
Comparison of the expression (**A**) KIF11 and (**B**) KIF14 in tumor and normal tissues in endometrial samples.

This study included the analysis of the expression of proteins KIF11 and KIF14 in endometrial cancer specimens and non-neoplastic endometrial tissues. Figure 1A–F displays representative images of the immunoexpression of the proteins analyzed. The predominant expression pattern of KIF11 was found to be localized to the cytoplasm, while KIF14 was predominantly expressed in the nucleus, but also in the cytoplasm of cells. Among 92 endometrial cancers, 38 (41.30%) demonstrated KIF11 immunoreactivity, while normal endometrial tissue showed a consistent low staining pattern. KIF11 expression was evidently upregulated in endometrial cancer tissues compared with non-neoplastic tissues in the tumor area (*p* < 0.0001; Figure 2A). According to the established division, 38 specimens (41.30%) of tumor tissue were characterized by high and 54 (58.70%) by low KIF11 expression. Our analysis revealed that the expression of KIF11 did not show any significant correlation with the clinical-pathological features of the cohort. Immunostaining of KIF14 was detected in all cases (100%) of endometrial cancers and non-malignant endometrial tissues. We found a statistically significant decrease in the expression score of KIF14 when comparing control and tumor tissues (*p* < 0.0001; Figure 2B). In turn, overexpression of KIF14 was noted in 58 samples (63.04%), while low expression was found for 34 patients (36.96%). Our investigation revealed a significant correlation between KIF14 expression and lymphovascular invasion (*p* = 0.0253). The expression status of KIF11 and KIF14 in relation to clinicopathological features are shown in Table 1.

### 3.3. Association Between KIF11, KIF14, and Combined KIF11/KIF14 Expression and Patient Survival

**Figure 3:**
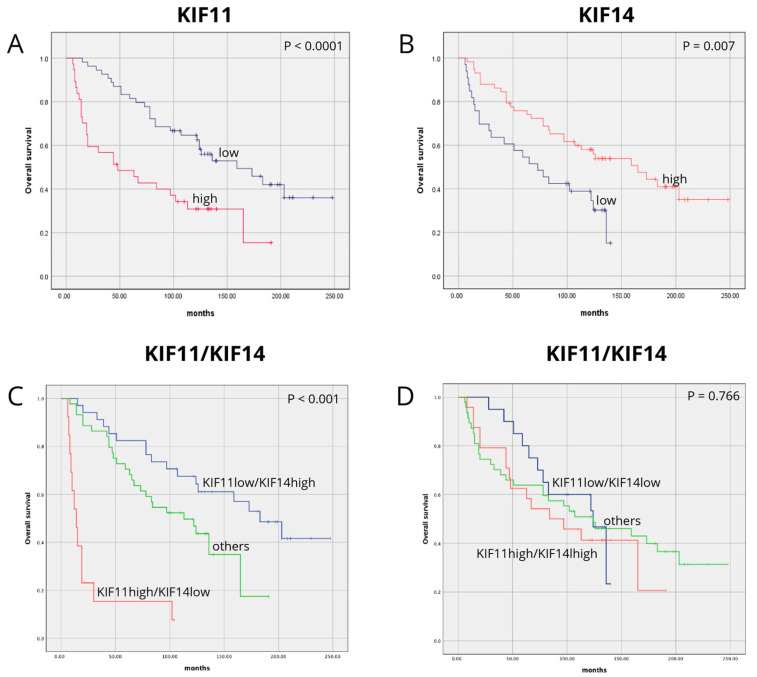
Overall survival analysis according to protein expression of (**A**) KIF11, (**B**) KIF14, (**C**) KIF11low/KIF14high vs. KIF11high/KIF14low, and (**D**) KIF11low/KIF14low vs. KIF11high/KIF14high in EC.

A Kaplan–Meier survival analysis indicated that EC patients with high KIF11 levels had lower overall survival (OS) rates than those with KIF11 low expression levels (47 vs. 159 months, *p* < 0.0001; Figure 3A). A univariate analysis showed that high KIF11 expression was significantly associated with a worse survival prognosis (HR 2.645, 95% CI 1.54–4.54, *p* < 0.001; Table 2), and it remained a prognostic factor for deteriorations of OS in a multivariate analysis after adjustment for age, histological grade, pT status, pN status, pM status, and lymphovascular invasion (HR 2.327, 95% CI 1.35–4.02, *p* = 0.002; Table 2). In turn, EC patients with low KIF14 expression tended to survive for shorter periods of time than those with high expression (73 vs. 158 months, *p* = 0.007; Figure 3B). The univariate analysis showed that low KIF14 expression was significantly related to poorer survival prognosis (HR 2.106, 95% CI 1.21–3.67, *p* = 0.008; Table 2), and it remained a prognostic factor in the multivariate analysis (HR 2.12, 95% CI 1.2–3.94, *p* = 0.016; Table 2). The results of the univariate and multivariate analysis are reported in Table 2.

After establishing the significance of the tested factors as individual prognostic markers, we further examined the impact of their combined expression on OS. Kaplan–Meier analysis demonstrated that patients with EC exhibiting a KIF11highKIF14low expression pattern had the worst OS. In contrast, those with the KIF11highKIF14low expression profile showed significantly longer OS (14 vs. 183 months, *p* < 0.001; Figure 3C). On the other hand, patients with EC exhibiting a KIF11high/KIF14high vs. KIF11low/KIF14low expression profile showed not significantly associated with OS (84 vs. 124 months, *p* = 0.766; Figure 3D). In the univariate (HR 4.663, 95% CI 2.34–9.30, *p* < 0.001; Table 3) and the multivariate (HR 3.769, 95% CI 1.81–7.87, *p* < 0.0001; Table 4) analyses, the combined KIF11^high^KIF14^low^ was a significantly poor prognostic factor for OS with a particularly high hazard ratio when compared to each marker as a single indicator.

## 4. Discussion

EC is one of the most prevalent gynecological neoplasms worldwide, and its incidence continues to rise. The advanced stage of the disease is associated with high mortality rates and frequent failures in response to adjuvant therapies. Therefore, it is crucial to identify effective prognostic indicators to predict the outcome of EC and provide new therapeutic targets. In this context, we investigated the expression and prognostic significance of KIF11 and KIF14 proteins in EC using immunohistochemistry on our own cohort. Nevertheless, to the best of our knowledge, apart from the current report, there are no studies reporting the clinical value of KIF11 and KIF14 protein in survival stratification of EC patients.

In recent years, many studies have been carried out to assess the role and level of expression of KIF11, and these findings indirectly confirm that KIF11 may play an essential function in carcinogenesis. KIF11 has been shown to activate the Wnt/β-catenin and mevalonate metabolism pathways, driving cancer cell proliferation. It also interacts with VEGF, facilitating angiogenesis. Furthermore, KIF11 plays a role in the PI3K/AKT, MAPK/ERK, and NF-κB/JNK signaling pathways, contributing to cancer cell invasion and promoting the epithelial–mesenchymal transition (EMT) process. [22]. According to previous studies, overexpression of KIF11 was shown in breast cancer [23], epithelial ovarian cancer [24], clear cell renal cell carcinoma [25], colorectal cancer [26], hepatocellular carcinoma [27], pancreatic adenocarcinoma [28], oral cancer [29], and lung cancer [30]. In agreement with these reports, we found that KIF11 protein was significantly upregulated in EC tissues compared with control endometrial tissues. In a study, Guo et al. analyzed immunohistochemical staining for KIF11 in the HPA database and suggested that the expression level of KIF11 was higher in different pan-cancer compared with normal tissues [31]. In our study, a distinct cytoplasmic KIF11 staining pattern was observed, like the study by Klimaszewska-Wiśniewska et al. (patients with pancreatic cancer) [28] and Neska-Długosz et al. (patients with colorectal cancer) [26]. Moreover, Iwakiri et al. highlighted that the regulatory mechanism of KIF11 localization varies among different cells and tissues [32]. Some previous reports from other authors have found that high KIF11 expression was significantly correlated with aggressive clinical characteristics of cancer [23,24,25,27,30], but we did not observe any significant relationships. However, one limitation of our study was the uneven distribution of clinicopathological data within the cohort, which could have potentially impacted the analysis and interpretation of the results. In our analysis, we observed a trend toward increased KIF11 expression with higher tumor grading, suggesting a potential association between KIF11 and more aggressive tumor phenotypes. While this trend was not statistically significant, it may indicate that KIF11 plays a role in tumor progression and dedifferentiation. Given that KIF11 is involved in key oncogenic pathways, including Wnt/β-catenin and PI3K/AKT, its upregulation in high-grade tumors could contribute to enhanced proliferative and metastatic potential. These findings align with previous reports based on TCGA data indicating that KIF11 overexpression is linked to poor prognosis in EC [33]. However, our data suggest that KIF11 expression is regulated by factors beyond traditional histopathological characteristics such as tumor grade or patient age. Further studies are needed to elucidate the molecular mechanisms underlying KIF11-mediated tumor progression and to determine its potential as a therapeutic target.

Regarding prognostic significance, we observed that high KIF11 protein expression was significantly associated with worse patient survival. Furthermore, we showed that high KIF11 protein expression was an independent unfavorable prognostic factor for the overall survival of patients. This finding was consistent with those made in previous investigations that showed the association between high KIF11 and poor survival in patients [23,24,25,26,27,29,30]. Previously, KIF11 was identified by Zang et al. as one of the key genes in EC by bioinformatics analyses [34]. In the cited study, according to the enrichment analysis of DEGs, KIF11 was associated with ‘mitotic cell cycle process’, ‘cell cycle process’, and ‘mitotic cell cycle’ [34]. According to previous studies, loss of KIF11 activity due to immunodepletion, mutation, RNA interference, or inhibition can cause cell cycle arrest and apoptosis, leading to cell death [35,36,37,38,39]. Furthermore, Castillo et al. demonstrated that transgenic mice overexpressing KIF11 showed a high propensity to develop various malignancies [16]. Considering the results shown, it is suggested that KIF11 may act as an oncogene throughout cancer progression. Therefore, further studies are essential to verify the function of KIF11 in EC.

KIF14 is a microtubule-associated motor protein from the kinesin-3 family, playing a crucial role in cytokinesis through its internal motor domain with microtubule-dependent ATPase activity. Additionally, it is involved in various biological processes, including cell proliferation, intracellular transport, and apoptosis [40]. KIF14 is referred to as an “oncogenic kinesin” and is a kinesin that has been shown to play a crucial role in the pathogenic process of various malignancies. Despite extensive research, its role and function in endometrial cancer remain unclear; to the best of our knowledge, this study is the first to evaluate the clinical significance of KIF14 in EC patients. In our study, the expression level of KIF14 protein was significantly lower in EC samples than in non-neoplastic tissues. Similar relationships have been observed in earlier reports of colorectal cancer [26] and lung adenocarcinoma [41]. In our study, KIF14 expression was predominantly localized in the nucleus and cytoplasm of endometrial cancer cells, aligning with the findings of Klimaszewska-Wiśniewska et al. in pancreatic cancer [28] and Gerashchenko et al. in breast cancer [42]. Contrary to our results, previous studies have shown that KIF14 expression was significantly increased in gastric cancer tissues [43], hepatocellular carcinoma [44], glioma [45], pancreatic adenocarcinoma [28], and medulloblastoma [46], suggesting that its precise role may depend on the tumor type and/or context.

In the present study, we identified a significant association between reduced KIF14 expression and the presence of lymphovascular space invasion (LVSI) in endometrial cancer. This finding is consistent with previous reports indicating that decreased KIF14 expression correlates negatively with aggressive tumor phenotypes, including enhanced invasive capabilities in lung cancer cell lines and Matrigel or perineural invasion in pancreatic cancer [41]. These findings indicate that, in some cancers, KIF14’s interaction with adhesion molecules may induce apoptosis, suppress Akt signaling, and inhibit epithelial–mesenchymal transition (EMT), thereby exerting tumor-suppressive effects.

The Kaplan–Meier analysis in our study demonstrated that patients with EC exhibiting low KIF14 expression tend to have poorer overall survival (OS). This finding aligns with emerging evidence from other malignancies, suggesting that KIF14 may serve as a prognostic marker with potential clinical relevance. For example, studies in colorectal cancer have shown that low KIF14 expression is associated with advanced disease and poor prognosis, supporting its role as a favorable prognostic marker in certain tumor contexts [26,46]. Similarly, in pancreatic adenocarcinoma, KIF14 downregulation has been linked to increased tumor invasiveness and poorer survival outcomes [28]. Interestingly, KIF14’s role as a prognostic indicator is also evident in specific brain tumors, such as WNT-subgroup gliomas, where higher KIF14 expression correlates with improved survival [46]. These findings suggest that the prognostic value of KIF14 is likely tumor-type-specific and may depend on the underlying molecular pathways and cellular contexts unique to each malignancy. Furthermore, the multivariate analysis revealed that the low expression of KIF14 was a statistically significant risk factor affecting overall survival in EC patients. Our results suggest that KIF14 acts as a tumor suppressor, which agrees with previous research in which it was shown that overexpression of KIF14 reduced cell proliferation in lung cancer [41,47]. Unlike KIF11, the expression of KIF14 did not exhibit a significant association with tumor grade in our study. Despite its established role as a key regulator of cytokinesis and cell cycle progression, KIF14 expression remained relatively stable across different histological grades, suggesting that its regulation in EC may follow a distinct pattern independent of tumor differentiation. Interestingly, KIF14 has been reported to act as either an oncogene or a tumor suppressor depending on the cancer type, and its precise role in EC remains unclear. Our results contribute to the growing body of evidence that KIF14 serves as a prognostic biomarker in endometrioid carcinoma.

In the subsequent analyses, we examined the possible effect of the combined expression of KIF11 and KIF14 on the overall survival of EC patients. The Kaplan–Meier analysis revealed that the worst OS was observed in patients whose ECs expressed KIF11highKIF14low. The set of two markers proved to be a strong independent prognostic factor and better predicted patient survival than each marker separately. In the other studies in breast cancer, overexpression of KIF11 and KIF14 has been associated with worse overall survival, indicating their potential as prognostic biomarkers [48]. Our findings in endometrial cancer, where high KIF11 expression combined with low KIF14 expression correlates with shorter overall survival, align with these studies. This suggests that dysregulation of kinesin family members may play a significant role in tumor progression across various cancer types. Additionally, in colorectal cancer (CRC), studies have demonstrated that altered expression levels of KIF11 and KIF14 are associated with patient outcomes. Specifically, increased KIF11 expression and decreased KIF14 expression correlate with poorer overall survival, suggesting that the combined expression of these proteins can serve as an independent prognostic marker in CRC [26]. Similarly, in pancreatic adenocarcinoma, elevated KIF11 expression has been linked to adverse clinical outcomes. Research indicates that higher levels of KIF11 are associated with increased tumor aggressiveness and reduced overall survival in pancreatic cancer patients. These findings align with our observations in endometrial cancer, where high KIF11 expression combined with low KIF14 expression correlates with shorter overall survival [28]. The consistent association across multiple cancer types underscores the potential of KIF11 and KIF14 as prognostic biomarkers and highlights the importance of further investigating their roles in tumor progression and patient outcomes. Further research into the combined expression profiles of KIF11 and KIF14 could enhance prognostic assessments and inform targeted therapeutic strategies.

Understanding the mechanisms by which KIF11 and KIF14 influence cancer progression could inform the development of targeted therapies aimed at modulating their expression or function, potentially improving prognoses for patients with various malignancies.

## 5. Conclusions

Our findings demonstrate a strong and consistent association between KIF11 and KIF14 expression and endometrioid carcinoma (EC), emphasizing their role in tumor progression. The results suggest that the expression patterns of these proteins may serve as useful prognostic markers in EC. However, while kinesins have been implicated in cancer progression, further studies are required to elucidate the precise molecular mechanisms underlying KIF11 and KIF14 expression in EC. Future research should focus on validating these findings in larger cohorts and exploring their biological significance in the context of tumor development and progression.

## Figures and Tables

**Table 1:** Immunohistochemical expression of KIF11 and KIF14 proteins and their relationship with clinicopathological features of EC patients.

		KIF11			KIF14		
Variables	Number	High	Low	*p* Value	High	Low	*p* Value
		*n* = 38	*n* = 54		*n* = 58	*n* = 34	
Age							
≤60	30	10	20	0.3673	21	9	0.3669
>60	62	28	34		37	25	
Histologic grade							
G1	7	1	6	0.1026	4	3	0.7181
G2	60	23	37		36	24	
G3	25	14	11		18	17	
pT status							
T1	54	22	32	0.7133	34	20	0.6209
T2	22	11	11		14	8	
T3	13	4	9		10	3	
T4	3	1	2		0	3	
pN status							
N0	79	31	48	0.3713	50	29	>0.9999
N1	13	7	6		8	5	
pM status							
M0	84	33	51	0.2675	55	29	0.1403
M1	8	5	3		3	5	
Lymphovascular invasion							
Present	16	9	7	0.7800	6	10	0.0253
Absent	76	47	29		52	24	
FIGO							
I	48	19	29	0.3259	30	18	0.3918
II	18	9	9		12	6	
III	18	5	13		13	5	
IV	8	5	3		3	5	

**Table 2:** Univariate and multivariate Cox proportional hazards models for overall survival of EC patients.

Variable	Univariate Analysis of Own Cohort				Multivariate Analysis of Own Cohort: KIF11				Multivariate Analysis of Own Cohort: KIF14			
	HR	95% CI		*p* Value	HR	95% CI		*p* Value	HR	95% CI		*p* Value
		Lower	Upper			Lower	Upper			Lower	Upper	
KIF11	2.645	1.540	4.542	<0.0001	2.327	1.346	4.023	0.002	-	-	-	-
KIF14	2.106	1.210	3.665	0.008	-	-	-	-	2.127	1.150	3.937	0.016
age	3.561	1.721	7.367	0.001	3.227	1.473	7.068	0.003	3.167	1.472	6.813	0.003
grade	2.656	1.538	4.585	<0.0001	2.478	1.300	4.725	0.006	2.963	1.482	5.921	0.002
pT	1.273	0.747	2.170	0.374	0.704	0.366	1.353	0.292	0.704	0.362	1.369	0.300
pN	1.974	1.017	3.832	0.044	1.624	0.686	3.845	0.270	1.858	0.739	4.670	0.188
pM	1.489	0.588	3.768	0.401	0.593	0.184	1.915	0.383	0.286	0.074	1.104	0.069
LVSI	5.419	2.873	10.221	<0.0001	3.529	1.785	6.979	<0.0001	3.419	1.637	7.140	0.001

**Table 3:** Univariate analysis of prognostic factors by the Cox proportional hazard model for combined expression.

Variable	Univariate Analysis of Own Cohort			
	HR	95% CI		*p* Value
		Lower	Upper	
Others				
KIF11^low^KIF14^high^	0.515	0.267	0.996	0.048
KIF11^high^KIF14^low^	4.663	2.339	9.296	<0.0001

**Table 4:** Multivariate analysis of prognostic factors by the Cox proportional hazard model for combined expression.

Variable	Multivariate Analysis of Own Cohort: KIF11/KIF14			
	HR	95% CI		*p* Value
		Lower	Upper	
Others				
KIF11^low^KIF14^high^	0.570	0.284	1.143	0.113
KIF11^high^KIF14^low^	3.769	1.806	7.869	<0.0001
age	2.780	1.263	6.119	0.011
grading	2.636	1.343	5.172	0.005
pT	0.675	0.347	1.313	0.247
pN	2.102	0.840	5.259	0.112
pM	0.365	0.103	1.292	0.118
LVSI	3.506	1.702	7.220	0.001

## Data Availability

Publicly available datasets were analyzed in this study. These data can be found here: https://www.proteinatlas.org (accessed on 25 September 2021). Our own data presented in this study are available on request from the corresponding author. The data are not publicly available due to ethical restrictions.

## Supplementary Materials

The following supporting information can be downloaded at: https://www.mdpi.com/article/10.3390/cancers17050804/s1, Table S1: Clinicopathological characteristics of 92 patients with endometrioid carcinoma from our cohort.
