# Supplementary material for: KIF11 and KIF14 Are a Novel Potential Prognostic Biomarker in Patients with Endometrioid Carcinoma
Source: Cancers (Basel). 2025 Feb 26;17(5):804. doi: 10.3390/cancers17050804 (PMC11898751; doi:10.3390/cancers17050804)
Supplement: Supplementary file 1 [file cancers-17-00804-s001.zip › cancers-3447980-supplementary.pdf]

Supplementary Materials

Supplementary Table S1. Clinicopathological characteristics of 92 patients with endometrioid carcinoma from our cohort.

Variables	Number (%)
Age	
≤60	30 (32.60)
>60	62 (67.40)
Histologic grade	
G1	7 (7.61)
G2	60 (65.22)
G3	25 (27.17)
pT status	
T1	54 (58.70)
T2	22 (23.91)
T3	13 (14.13)
T4	3 (3.26)
pN status	
N0	79 (85.87)
N1	13 (14.13)
pM status	
M0	84 (91.30)
M1	8 (8.70)
Lymphovascular invasion	
Present	16 (17.39)
Absent	76 (82.61)
FIGO	
I	48 (52.16)
II	18 (19.57)
III	18 (19.57)
IV	8 (8.70)
